# Upregulation of Pluripotency Markers in Adipose Tissue-Derived Stem Cells by miR-302 and Leukemia Inhibitory Factor

**DOI:** 10.1155/2014/941486

**Published:** 2014-07-23

**Authors:** Masoumeh Fakhr Taha, Arash Javeri, Sara Rohban, Seyed Javad Mowla

**Affiliations:** ^1^Department of Medical Biotechnology, National Institute of Genetic Engineering and Biotechnology (NIGEB), Pajoohesh Boulevard, P.O. Box 14965-161, Tehran 1417863171, Iran; ^2^Department of Molecular Genetics, Faculty of Biological Sciences, Tarbiat Modares University, P.O. Box 14115-154, Tehran, Iran

## Abstract

The expression pattern of pluripotency markers in adipose tissue-derived stem cells (ADSCs) is a subject of controversy. Moreover, there is no data about the signaling molecules that regulate these markers in ADSCs. In the present study, we studied the roles of leukemia inhibitory factor (LIF) and miR-302 in this regard. Freshly isolated mouse ADSCs expressed hematopoietic, mesenchymal, and pluripotency markers. One day after plating, ADSCs expressed OCT4 and Sox2 proteins. After three passages, the expression of hematopoietic and pluripotency markers decreased, while the expression of mesenchymal stem cell markers exhibited a striking rise. Both supplementation of culture media with LIF and transfection of the ADSCs with miR-302 family upregulated the expression levels of *OCT4*, *Nanog*, and *Sox2* mRNAs. These findings showed that mouse adipose tissue contains a population of cells with molecular resemblance to embryonic stem cells, and LIF and miR-302 family positively affect the expression of pluripotency markers.

## 1. Introduction

White adipose tissue represents a rich source of stem cells with potential applications in basic and clinical research. Adipose tissue-derived stem cells (ADSCs) can be harvested from patients by a simple and minimally invasive method. They can be easily cultured and rapidly propagated [1]. Previous studies have characterized ADSCs as a lineage with mesenchymal stem cell (MSC) nature [2–5]. Peroni and colleagues [6] showed that bone marrow-derived mesenchymal stem cells (BM-MSCs) and ADSCs have a virtually identical transcriptional profile for stemness-related genes. Moreover, both cells express embryonic stem (ES) cell-specific genes, including* OCT4*,* UTF1*, and* Nodal*. This finding was against a previous report by Case and colleagues [7]. In fact, there is significant controversy around the expression of pluripotency markers in the ADSCs. In addition, there is no data about the signaling molecules that regulate these markers in the ADSCs.

In the present study, freshly isolated and third-passaged ADSCs were examined for the expression of some hematopoietic, mesenchymal, and pluripotency markers at mRNA and protein levels. Moreover, the effects of leukemia inhibitory factor (LIF) and ES cell-specific miRNA, miR-302 family [8], on the expression of pluripotency markers in mouse ADSCs were investigated.

## 2. Materials and Methods

### 2.1. Isolation and Culture of Mouse ADSCs

ADSCs were isolated from the inguinal adipose tissue of 10–12-week-old Balb/c mice using 2 mg/mL collagenase A (Roche, Mannheim, Germany) digestion, as previously described [9]. Isolated cells were counted and plated at 5 × 10^4^ cells/mL seeding density in 6 cm tissue culture plates and cultured in Dulbecco's modified eagle's medium (DMEM, Gibco, Life Technologies, USA) with 20% fetal bovine serum (FBS, Gibco). Cells were passaged after reaching 80–90% confluency. The culture medium was changed every 2 days.

### 2.2. Characterization of the ADSCs

For characterization of the expression of cell surface markers, third-passaged ADSCs were labeled with phycoerythrin (PE) conjugated rat monoclonal anti-mouse CD105, CD29, and CD31 and fluorescein isothiocyanate (FITC) conjugated rat anti-mouse CD11b and CD45. The cells stained with FITC- or PE-labeled rat anti-mouse IgG were considered as negative controls. After fixation with 1% paraformaldehyde, the cells were examined by an Attune Acoustic Focusing Cytometer (Applied Biosystems, Life Technologies, USA) and were analyzed using FlowJo 7.6.1 software (Tree Star, Inc., Ashland, USA).

### 2.3. The Effect of LIF on the Expression of Pluripotency Markers in Cultured ADSCs

Freshly isolated stromal vascular fraction (SVF) was cultured in DMEM with 20% FBS and 1000 IU/mL LIF (Chemicon, ESGRO). The same medium was used throughout the next passages. The ADSCs cultured at the presence or absence of LIF were compared for the expression of pluripotency markers,* OCT4*,* Sox2*, and* Nanog*.

### 2.4. Transfection of Third-Passaged ADSCs with pEGFPC1-miR-302 Vector

Third-passaged ADSCs were transfected with the pEGFPC1-miR-302 or mock vectors (ParsGenome). In the pEGFPC1-miR-302 vector, the EGFP coding sequence and the precursor of miR-302a/b/c/d have their own CMV promoter (Figure 6(a)).

Transfection of the ADSCs was performed using Lipofectamine 2000 (Invitrogen, Life Technologies). After transfection, the cells were incubated at 37°C in a CO_2_ incubator. After 8–10 hours, medium of transfected cells was replaced with fresh medium and 1000 IU/mL LIF. After 48 hours, ADSCs were harvested and assessed for the expression of pluripotency markers.

For antibiotic selection of transfected ADSCs, 48 h after transfection, medium was changed and 200 mg/mL G418 (Roche) was added. Medium and G418 were replaced every day until nonresistant cells were cleared from the cultures.

### 2.5. Reverse Transcription-Polymerase Chain Reaction (RT-PCR)

Total RNA was extracted using High Pure RNA Isolation Kit (Roche), according to the manufacturer's instructions. 1 *μ*g of total RNA was transcribed into cDNA using oligo-dT primers and RevertAid H Minus MMuLV Reverse Transcriptase (Fermentas, Thermo Fisher Scientific Inc., USA). Polymerase chain reaction (PCR) was performed using specific primers. Primer sequences, and the size of the PCR products are shown in Table 1. PCR products were size fractionated by 1.5% agarose gel electrophoresis.

### 2.6. Quantitative Real-Time PCR

For quantitative real-time PCR (qPCR) analysis, specific primers for* OCT4*,* Sox2*,* Nanog*, and* AFP* genes at 100 nM final concentration were used (Table 1). *β*-Tubulin 5 (*Tubb5*) was selected as the internal reference gene. qPCR was performed using RealQ PCR Master (Ampliqon A/S, Denmark) on a Rotor-Gene 6000 (Corbett Research, Qiagen) real-time analyzer with cycling parameters of 95°C for 15 min, then 45 cycles of 15 seconds at 95°C, and 40 seconds at 61°C and a final melt analysis. Comparative quantitation was performed between selected groups using REST 2009 (Relative Expression Software Tool, Qiagen, GmbH). At least four biologic replicates of each group were included in the qPCR experiments.

### 2.7. Immunofluorescence and Western Blot

For immunostaining, cells were fixed using 4% paraformaldehyde, permeabilized by 0.5% Triton X-100 (Sigma), blocked with 10% goat serum (Gibco), and incubated with primary and secondary antibodies for 45 min at 37°C. Antibodies used in this study included monoclonal antibodies for OCT4 (C-10, sc-5279; Santa Cruz Biotechnology), Sox2 (MAB4343; Millipore), and anti-mouse FITC-conjugated IgG antibody (F9006; Sigma). Preparations were examined and photographed using an inverted fluorescence microscope (Nikon, Eclipse TE 2000U, Japan).

For western blot analysis, ADSCs were homogenized in ice-cold RIPA lysis buffer and centrifuged at 12,000 rpm for 15 minutes at 4°C. The supernatant was collected, and the protein concentration was determined using the Coomassie Blue assay. For each sample, 50 *μ*g of protein was separated using SDS-PAGE and transferred to PVDF membranes. The membranes were blocked with 5% nonfat dried milk in Tris-buffered saline containing 0.1% Tween-20 (TBST) for 1 hour and incubated with the primary antibody against OCT4A (C-10, sc-5279; Santa Cruz Biotechnology) overnight at 4°C. Then, the membrane was incubated with goat anti-mouse HRP-conjugated secondary IgG for 1 hour at room temperature and immunoreactive bands were detected using ECL Plus Detection Kit (Amersham Bioscience).

## 3. Results

### 3.1. Isolation and Characterization of ADSCs

**Figure 1:**
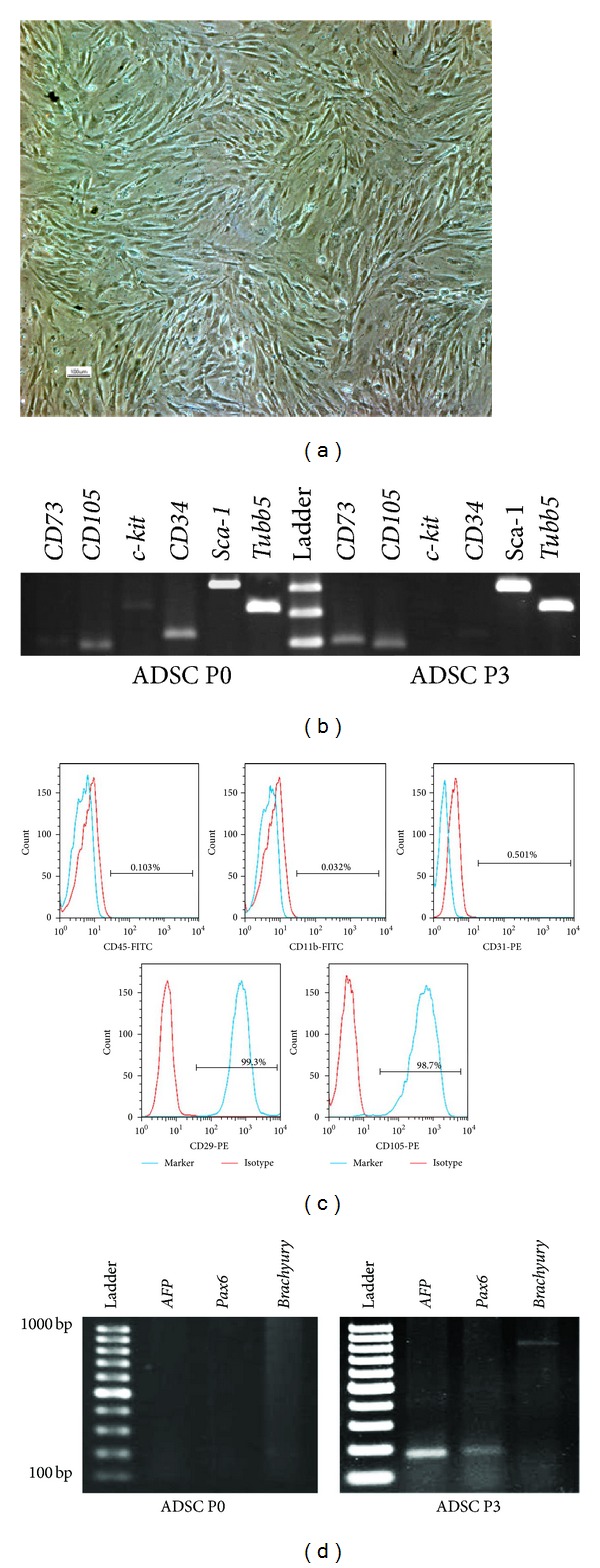
(a) Third-passaged ADSCs with a homogenous fibroblast-like morphology, (b) expression of stem cell marker, Sca-1, mesenchymal stem cell markers, CD73 and CD105, and hematopoietic cell markers, CD34 and c-Kit in the freshly isolated (ADSC P0) and third-passaged ADSCs (ADSC P3), (c) flow cytometry histograms of third-passaged ADSCs for hematopoietic (CD45 and CD11b), endothelial (CD31), and mesenchymal stem cell (CD29, and CD105) markers, and (d) expression of three germ layer markers,* AFP*,* Pax6*, and* Brachyury*, in the freshly isolated (ADSC P0) and third-passaged ADSCs (ADSC P3).

During the first day after plating, inguinal adipose tissue-derived stem cells adhered to the surface of tissue culture plates as a small population of polygonal or spindle-shaped cells. ADSCs propagated rapidly in vitro and developed a homogenous fibroblast-like morphology (Figure 1(a)). These cells were passaged three to four times per week, after reaching 80%–90% confluency.

Freshly isolated ADSCs expressed stem cell marker,* Sca-1*, mesenchymal stem cell markers,* CD73* and* CD105*, and hematopoietic cell markers,* CD34* and* c-Kit*, as detected by RT-PCR (Figure 1(b)). After three passages, the expression of* Sca-1*,* CD73*, and* CD105* mRNAs increased, while the expression of* CD34* mRNA decreased strikingly. Expression of* c-Kit* mRNA was barely detectable (Figure 1(b)).

Based on flow cytometry analysis, about 0.103%, 0.032%, and 0.501% of the third-passaged ADSCs were positive for CD45, CD11b, and CD31 proteins, respectively. The expression of CD29 and CD105 was detected in 99.3% and 98.7% of the third-passaged ADSCs (Figure 1(c)).

### 3.2. Expression of Germ Layer Markers in the ADSCs

As revealed by RT-PCR analysis,* Pax6*,* Brachyury*, and* AFP* were not detected in the freshly isolated ADSCs (Figure 1(d), ADSCs P0). After three passages, ADSCs always strongly expressed* AFP*, while* Pax6* and* Brachyury* were weakly expressed (Figure 1(d), ADSCs P3).

### 3.3. Expression of Pluripotency Markers in the ADSCs

**Figure 2:**
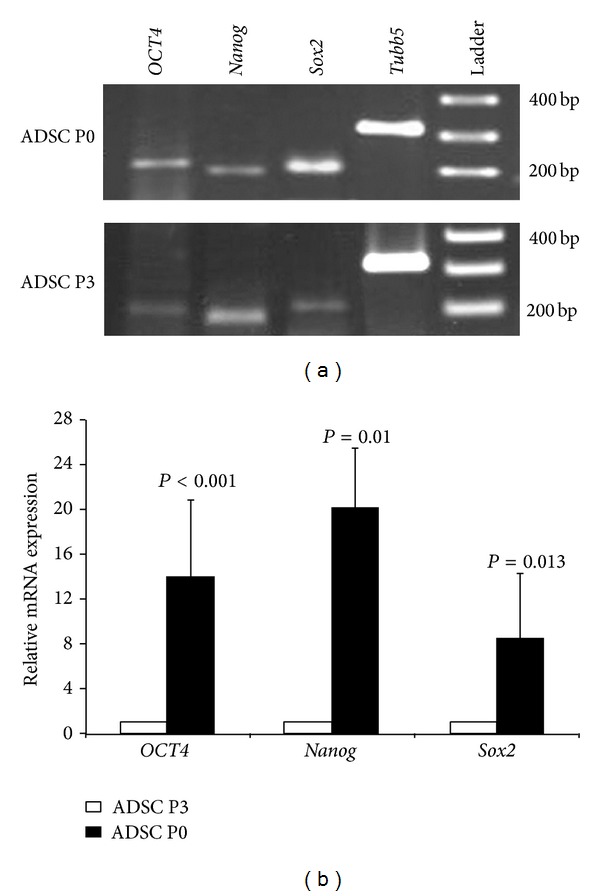
(a) RT-PCR analysis of the expression of pluripotency markers in the freshly isolated (ADSC P0) and third-passaged ADSCs (ADSC P3), (b) quantitative real-time PCR analysis for the expression of* OCT4*,* Sox2*, and* Nanog* mRNAs in the freshly isolated (ADSC P0) and third-passaged ADSCs (ADSC P3) using *β*-tubulin (*Tubb5*) mRNA level as an internal control. *P* < 0.05 was considered as significant.

Both freshly isolated ADSCs (ADSC P0) and third-passaged ADSCs (ADSC P3) expressed pluripotency markers,* OCT4*,* Sox2*, and* Nanog* (Figure 2(a)). However, the expression of* OCT4*,* Nanog*, and* Sox2*  mRNAs in the freshly isolated ADSCs was about 13.9, 20.1, and 8.4 times higher than the third-passaged ADSCs, respectively (Figure 2(b)).

**Figure 3:**
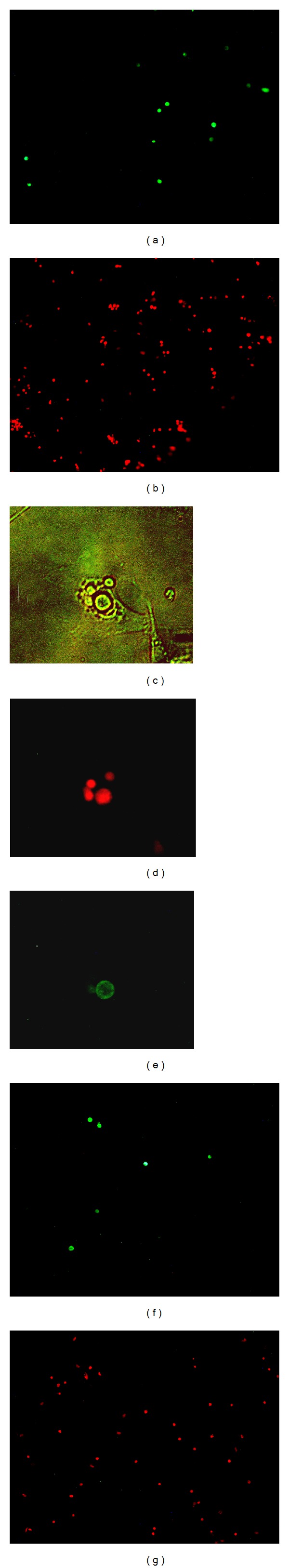
(a) Immunostaining of the freshly isolated ADSCs (ADSC P0) with anti-OCT3/4 antibody, (b) propidium iodide (PI) staining of the nuclei represented in (a). (c–e) Phase contrast, PI staining, and OCT3/4 immunostaining of the ADSC P0. Immunostaining was performed using a specific monoclonal antibody (sc-5279) against the OCT4A protein. Nuclear signal was detected in the ADSCs and recognized the OCT4A isoform. (f) Immunostaining of third-passaged ADSCs (ADSC P3) with anti-OCT3/4 antibody, (g) PI staining of the nuclei represented in (f).

**Figure 4:**
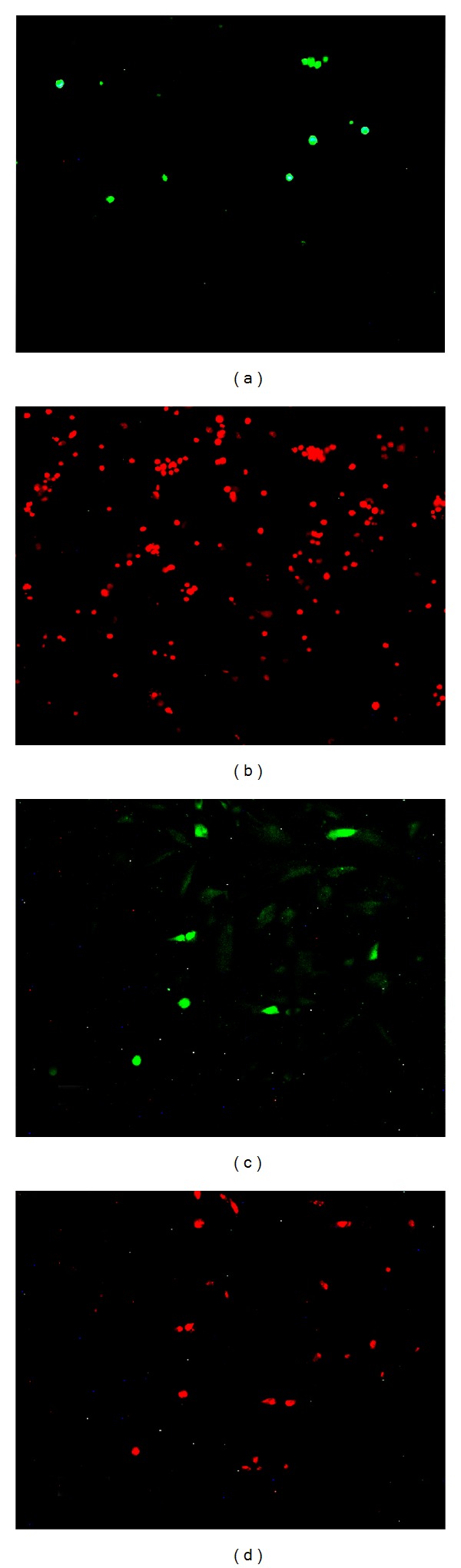
(a) Immunostaining of the freshly isolated ADSCs (ADSC P0) with anti-Sox2 monoclonal antibody, (b) propidium iodide (PI) staining of the nuclei represented in (a), (c) immunostaining of the third-passaged ADSCs (ADSC P3) with anti-Sox2 antibody, and (d) PI staining of the nuclei represented in (f).

In the present study, we performed RT-PCR analysis of* OCT4* expression using primers directed to amplify sequences from exon 1 to 2. Since exon 1 is unique for* OCT4A* transcript [10], this primer set is specific to* OCT4A*. Moreover, freshly isolated and third-passaged ADSCs were immunostained using a mouse monoclonal antibody (sc-5279; Santa Cruz Biotechnology) which recognizes amino acids 1–134 of OCT4A protein specifically [11, 12]. As shown in Figures 3(a) and 3(b), some freshly isolated ADSCs were positively immunostained with anti-OCT4 monoclonal antibody, with a nuclear localization (Figures 3(c)–3(e)). The number of OCT4-immunostained cells decreased after three passages (Figures 3(f) and 3(g)). Using anti-Sox2 antibody, the nuclei of some freshly isolated (Figures 4(a) and 4(b)) and third-passaged ADSCs (Figures 4(c) and 4(d)) were positively stained.

### 3.4. Effect of LIF on the Expression of Pluripotency Markers in the ADSCs

**Figure 5:**
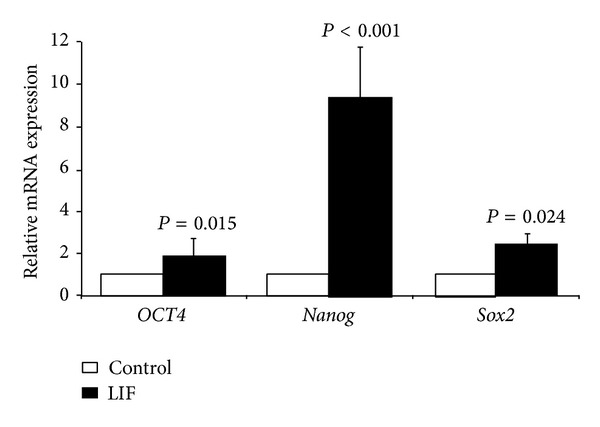
Quantitative real-time RT-PCR analysis for the expression of* OCT4*,* Sox2*, and* Nanog* mRNAs in the ADSCs cultured at the presence or absence of LIF;* Tubb5* mRNA level was used as an internal control. *P* < 0.05 was considered as significant.

Freshly isolated ADSCs were cultured and passaged at the presence or absence of 1000 IU/mL LIF. After three passages, the expression of pluripotency markers was compared between LIF and control groups by qPCR analysis. In comparison to ADSCs cultured without LIF,* OCT4*,* Nanog*, and* Sox2* expression was upregulated in the LIF-supplemented group by mean factors of 1.829, 9.341, and 2.432, respectively (Figure 5).

### 3.5. Transfection of the ADSCs with miR-302 Family

**Figure 6:**
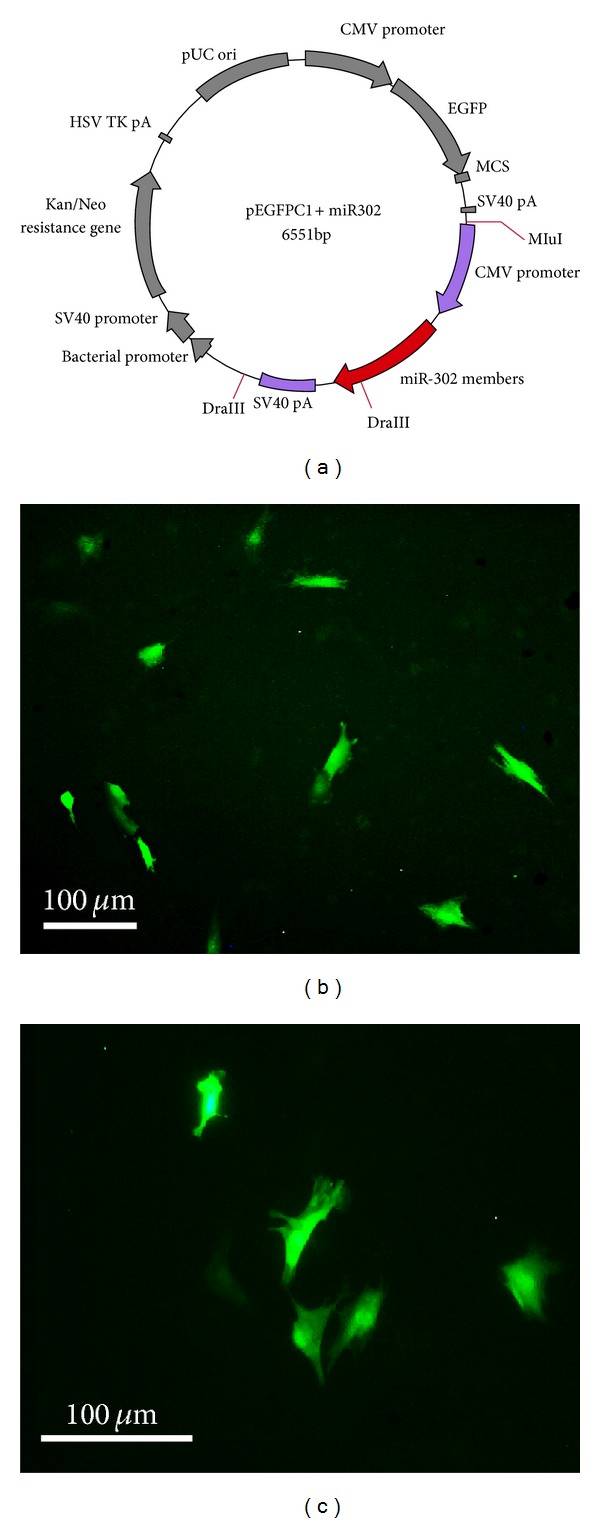
(a) The pEGFPC1-miR-302 vector, (b and c) expression of EGFP in the third-passaged ADSCs, 24 h after transfection with pEGFP-C1-miR-302 using Lipofectamine 2000.

Third-passaged ADSCs were cultured in 6 cm tissue culture plates to reach 80% confluency, and then transfection with miR-302 (Figure 6(a)) or mock vectors was performed using Lipofectamine 2000. 24 hours after transfection, ADSCs were observed under a fluorescent microscope for EGFP-positive cells (Figures 6(b) and 6(c)). Transfection efficiency of the ADSCs was about 7–10 percent.

**Figure 7:**
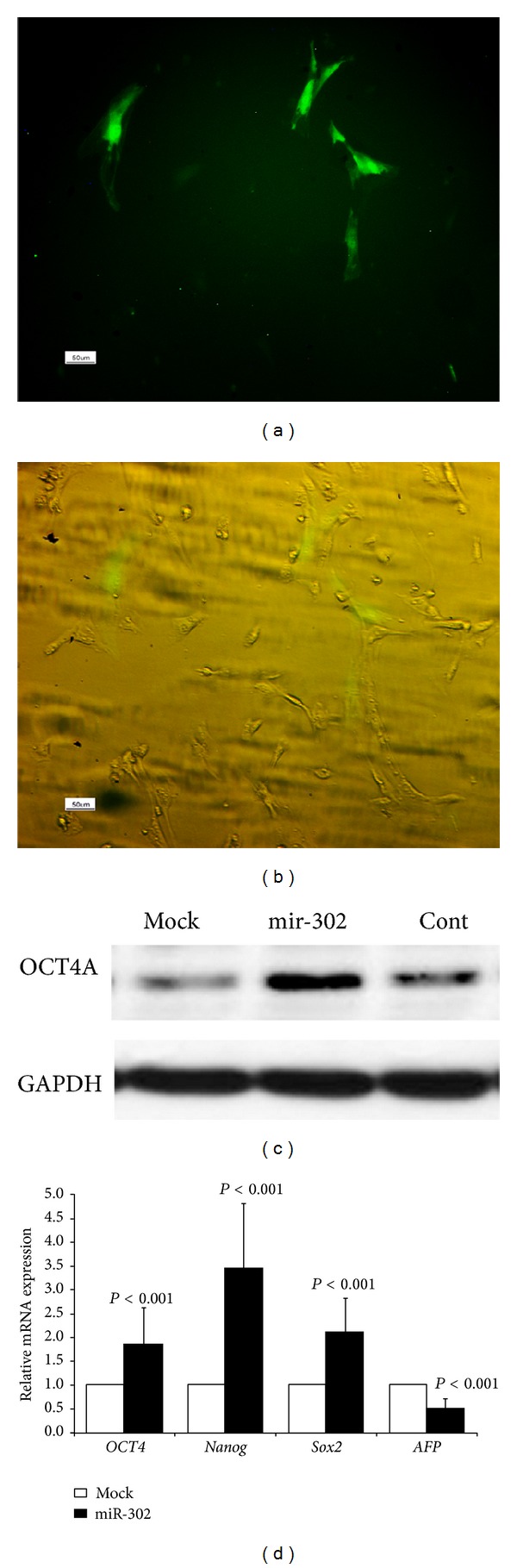
(a and b) Expression of EGFP in the third-passaged ADSCs, after three-day antibiotic selection, (c) western blot analysis for the expression of OCT4A protein in the transfected and control ADSCs, after 5-day antibiotic selection, and (d) quantitative real-time RT-PCR analysis for the expression of* OCT4*,* Nanog*,* Sox2*, and* AFP* mRNAs in the miR-302 group comparing to mock group, 48 hours after transfection.* Tubb5* mRNA level was used as an internal control. *P* < 0.05 was considered as significant.

After transfection, ADSCs were cultured in LIF-containing growth medium, and transfected ADSCs were selected by G418 treatment for 5 days. During this time, colony formation was not detected in the transfected cells (Figures 7(a) and 7(b)). After 5-day antibiotic selection, the expression of OCT4A protein was assessed in the transfected and control ADSCs by western blot.  Figure 7(c) shows a significant increase in OCT4A protein expression in the miR-302-transfected ADSCs.

### 3.6. Expression of Pluripotency Markers and AFP in the ADSCs after Transfection with miR-302 Family

48 hours after transfection, the expression levels of* OCT4*,* Nanog*, and* Sox2* in miR-302 group were 1.834, 3.442, and 2.101 times higher than the mock group, respectively (*P* < 0.001, Figure 7(d)). Since* Brachyury* and* Pax6* were rarely detected in the third-passaged ADSCs (Figure 1(d)), we only compared the expression level of* AFP* mRNA between the miR-302 and mock groups.* AFP* mRNA expression was downregulated in the miR-302 transfected ADSCs by a mean factor of 0.521 (*P* < 0.001) (Figure 7(d)).

## 4. Discussion

### 4.1. Expression of Pluripotency Markers in the Mouse ADSCs

Adipose tissue is a rich source of stem cells with molecular resemblance to BM-MSCs [6, 13]. These cells express several mesenchymal cell-specific genes, and after several passages they lose the expression of hematopoietic markers [4]. In this study, mesenchymal stem cell markers, CD105 and CD29, were expressed in 97% and 99% of the third-passaged ADSCs, respectively.

Recent findings support a close similarity between mesenchymal and embryonic stem cells [6]. So far, the expression of pluripotency markers has been shown in the cells isolated from porcine Wharton's jelly [14], equine umbilical cord blood [15, 16], mouse, equine, Rhesus and human BM-MSCs [13, 17, 18] and mouse, and Rhesus and human ADSCs [6, 13, 19, 20]. Nevertheless, it is still a source of controversy. Case and colleagues [7] did not detect the expression of* OCT4* and* Rex1* genes in the freshly isolated mouse ADSCs. In contrast to this report, we showed the expression of pluripotency markers,* OCT4*,* Sox2*, and* Nanog*, in the freshly isolated mouse ADSCs. This finding was in agreement with the previous reports by Izadpanah et al. [13] and Peroni et al. [6] in human.* OCT4*,* Sox2*, and* Nanog* are key factors that together with FoxD3 form an autoregulatory network and support or limit each other's expression. This interconnection is essential for maintaining the pluripotency and self-renewal properties of ES cells [21–23].

So far, three alternatively spliced variants have been reported for human* OCT4*, that is,* OCT4A*,* OCT4B*, and* OCT4B1* [12, 24, 25].* OCT4A* is localized within the nucleus and is responsible for stemness property of the pluripotent stem cells.* OCT4B* is localized within the cytoplasm of somatic cells, cell lines, and primary tumors, and while it cannot sustain self-renewal property of ES cells, it may respond to cell stress [11, 25, 26].* OCT4B1* is highly expressed in embryonic stem cells and embryonic carcinoma cells as a putative marker of stemness, and it is rapidly downregulated during differentiation [12].

According to some investigators, expression of alternatively spliced variants of* OCT4* and expression of* OCT4* pseudogenes can be two main sources of controversy [27]. Therefore, appropriate measures need to be taken in order to distinguish* OCT4A* isoform and to avoid confusion. In the present study, we included several considerations to detect the expression of* OCT4A* isoform in the mouse ADSCs, including treatment of all RNA samples with RNase-free DNase I, RT-PCR analysis using a forward primer specific to* OCT4A*, and immunostaining using a monoclonal antibody which specifically recognizes* OCT4A* protein [11, 12].

According to our immunostaining analyses,* OCT4A* protein was expressed and localized into the nuclei of the freshly isolated ADSCs, and the number of positively immunostained cells decreased after several passages. We obtained similar results for the expression of* Sox2* and* Nanog*, at mRNA and protein levels.

### 4.2. Effect of LIF on the Expression of Pluripotency Markers in the Cultured ADSCs

We supplemented the expansion medium of the ADSCs with 1000 IU/mL LIF and demonstrated that* OCT4*,* Nanog*, and* Sox2* expression can be maintained more efficiently at the presence of LIF. LIF belongs to the interleukin-6 cytokine family. LIF-pathway is a crucial element for regulation of self-renewal and maintenance of pluripotency in the ES and induced pluripotent stem (iPS) cells [28]. When LIF binds to LIF receptor, it activates the JAK/STAT3, PI3K/AKT, and SHP2/MAPK pathways [28]. These pathways converge to activate the specific gene expression pattern of mouse ES cells and to maintain the ES cells identity. It is interesting to note that BM-MSCs and ADSCs express both LIF and LIF receptor [6, 29, 30]. The role of LIF secretion by these stem cells is not fully understood, but it seems to mimic the role of LIF in undifferentiated propagation of mouse ES cells. LIF may be an important factor for preservation of pluripotent stem cells within the adipose tissue. Moreover, culture of SVF cells in LIF-containing media may be useful for preservation of pluripotency features in vitro.

### 4.3. The Effects of miR-302 on the Expression of Pluripotency Markers in the ADSCs

The key roles of miRNAs in maintenance, differentiation, and fate determination of mammalian ES cells have been studied during the last decade. In previous studies, a group of 31 miRNAs has been identified as a miRNA expression signature for human ES cells [8, 31, 32]. Moreover, Bar and colleagues [33] found that the most overexpressed miRNAs in undifferentiated human ES cells are miR-302b, miR-302c, miR-302d, miR-92b, miR-20b, miR-519d, miR-302a, miR-324-3p, miR-187, and miR-18b. Marson et al. [34] demonstrated that OCT4, Sox2, Nanog, and Tcf3 bind to the promoters of miR-302-367 cluster which is the most prevalent miRNAs in the ES cells [8, 35, 36]. As previously indicated, the maintenance of ES cell identity significantly depends on the regulatory role of miR-302 cluster [34]. Lin et al. [37–39] successfully used miR-302/367 cluster to reprogram human hair follicle cells, melanocytes, and some cancer cell lines to iPS cells, while similar experiments have also been performed on mouse and human fibroblasts later [40–42].

In the present study, third-passaged ADSCs were transfected with a recombinant vector expressing miR-302 cluster. 48 hours after transfection, the expression levels of* OCT4*,* Nanog*, and* Sox2* mRNAs in the miR-302 transfected ADSCs were significantly higher than the mock group, while the expression of* AFP* mRNA was reduced to about 50%. These findings demonstrated a significant upregulation in the expression level of ES cell-specific genes and inhibition of an endodermal marker. However, colony formation was not detected after transfection of the ADSCs with miR-302 family. This finding was in agreement with the recent studies by Hu et al. [43] and Anokye-Danso et al. [40] in ADSCs and embryonic fibroblasts, respectively. However, it has been shown that the expression of* OCT4* gene is not induced after transfection of mouse embryonic fibroblasts with miR-302s without miR-367 which is in contrast to our findings [40]. Significant upregulation of* OCT4*,* Sox2*, and* Nanog* mRNAs and OCT4A protein in the miR-302s-transfected ADSCs, despite the low transfection efficiency, shows that miR-302s play a role in regulating the expression of these genes independent of miR-367.

## 5. Conclusion

In summary, freshly isolated mouse ADSCs showed the expression of pluripotency markers at mRNA and protein levels. After three passages, the expression of pluripotency markers was eliminated, while the expression of mesenchymal cell-specific markers showed a striking enhancement. These findings show that white adipose tissue is containing a population of pluripotent stem cells with molecular resemblance to ES cells. Supplementation of the media with LIF led to a better preservation of pluripotency markers in the cultured ADSCs. Furthermore, transfection of the third-passaged ADSCs with miR-302 family resulted in upregulation of* OCT4*,* Nanog*, and* Sox2* gene expression. Our findings demonstrated that ADSCs can be used as a suitable source of cells for reprogramming studies using ES cell-specific miRNAs.

## Figures and Tables

**Table 1:** Primers used for RT-PCR and qPCR.

Genes	Forward	Reverse	Size (bp)	Accession number
*Sca-1*	5′-CTCTGAGGATGGACACTTCT-3′	5′-GGTCTGCAGGAGGACTGAGC-3′	404	NM_001271416
*CD34*	5′-ATGCAGGTCCACAGGGACACG-3′	5′-CTGTCCTGATAGATCAAGTAG-3′	220	NM_001111059
*c-kit*	5′-CCATGTGGCTAAAGATGAAC-3′	5′-ACTGCTGGTGCTCGGGTTT-3′	318	NM_001122733
*CD73*	5′-TCCTGGGCTACGATGCTATG-3′	5′-CCACAACCTCACCGCCAAC-3′	195	NM_011851
*CD105*	5′-AGCCTTACCTCTGGATACCG-3′	5′-AACGTCACCTCACCCCTTGT-3′	190	NM_007932
*OCT4A*	5′-TGTGGACCTCAGGTTGGACT-3′	5′-CTTCTGCAGGGCTTTCATGT-3′	201	NM_013633
*Sox-2*	5′-GCACATGAACGGCTGGAGCAACG-3′	5′-TGCTGCGAGTAGGACATGCTGTAGG-3′	206	NM_011443
*Nanog *	5′-GAGTGTGGGTCTTCCTGGTC-3′	5′-GAGGCAGGTCTTCAGAGGAA-3′	182	NM_028016
*Pax6 *	5′-TGCCCTTCCATCTTTGCTTG-3′	5′-TCTGCCCGTTCAACATCCTTAG-3′	178	NM_001244200
*Brachyury *	5′-ATGCCAAAGAAAGAAACGAC-3′	5′-AGAGGCTGTAGAACATGATT-3′	835	NM_009309
*AFP *	5′-TCGTATTCCAACAGGAGG-3′	5′-AGGCTTTTGCTTCACCAG-3′	174	NM_007423
*Tubb5*	5′-GGAACATAGCCGTAAACTGC-3′	5′-TCACTGTGCCTGAACTTACC-3′	317	NM_011655
